# An Elderly Smoker Presenting With Pulmonary Langerhans Cell Histiocytosis: An Unusual Demographic

**DOI:** 10.7759/cureus.14901

**Published:** 2021-05-08

**Authors:** Bilal Malik, Muhammad Ahmad, Atefeh Kalantary, Abhijeet Ghatol

**Affiliations:** 1 Internal Medicine, McLaren Health Care/Michigan State University, Flint, USA; 2 Pulmonary and Critical Care Medicine, McLaren Health Care, Flint, USA

**Keywords:** pulmonary, smoking tobacco, inspiratory dyspnea, lymph nodes, langerhans cell

## Abstract

Pulmonary Langerhans cell histiocytosis (PLCH) is a rare neoplastic condition that occurs almost exclusively in young smokers and presents with multiple solid and/or cystic nodules in a primarily upper lobe distribution on chest imaging. Frequently, suspicion arises incidentally on imaging performed for alternative reasons, such as lung cancer screening, but diagnosis requires biopsy studies. We describe an uncommon case of PLCH in an elderly female patient presenting with mild dyspnea and a significant history of smoking. Diagnosis was made with a biopsy of a new pulmonary nodule in her left lung found on lung cancer screening computed tomography (CT) scan. No gold standard therapy exists, and novel agents are being studied for future use. At present, she has been advised to quit smoking and is being followed with serial imaging studies to determine additional measures.

## Introduction

Pulmonary Langerhans cell histiocytosis (PLCH) is a rare neoplastic condition that occurs almost exclusively in young smokers and presents with multiple solid and/or cystic nodules in a primarily upper lobe distribution on chest imaging [1-3]. Around 90%-100% of the patients are heavy smokers. Adult Langerhans cell histiocytosis usually presents with single organ involvement, most often the lungs; however, extrapulmonary involvement is observed in less than 20% of the cases. Patients are usually asymptomatic on presentation but may have cough along with exertional dyspnea. Diagnosis is usually made incidentally on imaging performed for alternate reasons. CT scanning of the chest usually demonstrates characteristic findings including a combination of multiple cysts and nodules within the mid to upper lung zones predominantly [1-3]. This case exhibits the stereotypical findings of PLCH on imaging and biopsy studies. This particular patient is unique in that she falls well beyond the typical age demographic of 20-40 years [2], where this condition is most often expected, reinforcing the fact that we should consider atypical presentations in our differential diagnosis of lung nodules.

## Case presentation

Our patient is a 65-year-old female with a past medical history of chronic obstructive pulmonary disease (COPD), obstructive sleep apnea (OSA), type 2 diabetes mellitus, hypothyroidism, and atrial fibrillation. She did have a significant smoking history of 40 pack-years. She was initially seen in the pulmonology office for a routine lung cancer screening computed tomography (CT) scanning and complaints of dyspnea. On physical examination, the patient had good air entry bilaterally. Her chest was clear to auscultation with no wheezing or added sounds, and no lymphadenopathy was appreciated on palpation in the cervical, axillary, or clavicular areas. Laboratory studies, including complete blood count and complete metabolic panel, were within the normal limits. Pulmonary function tests performed in the office did not meet the criteria for obstruction. The forced expiratory volume over 1 second (FEV1) was 1.95 L (65% of predicted value) without reversibility, and lung volumes suggested mild restriction with mildly reduced diffusion capacity of carbon monoxide (DLCO). Three CT scans from 2010, 2015, and 2017 were reviewed, all of which revealed mosaic ground-glass appearance. At the previous time points, the mosaic appearance was mostly attributed to respiratory bronchiolitis associated with her smoking, and no further work-up was performed.

**Figure 1:**
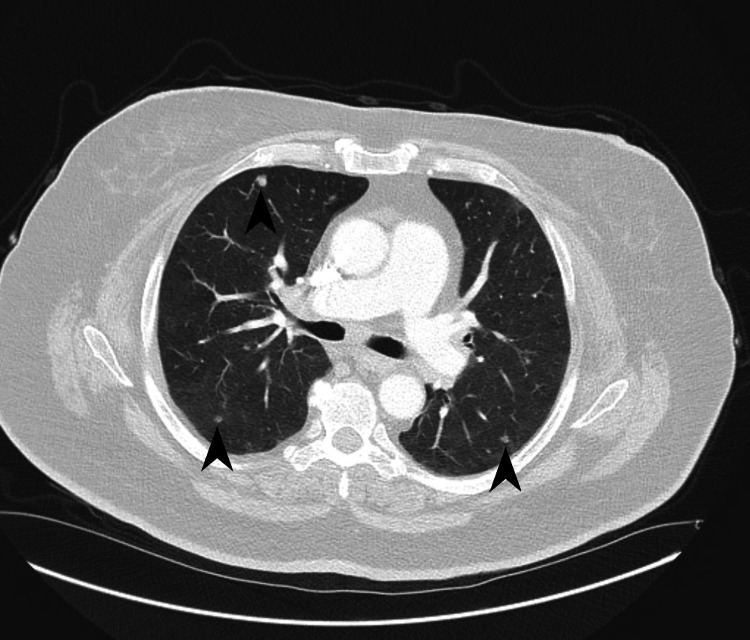
Transverse section of CT scan of the chest demonstrating multiple lung nodules in bilateral upper lobe distribution (black arrowheads).

**Figure 2:**
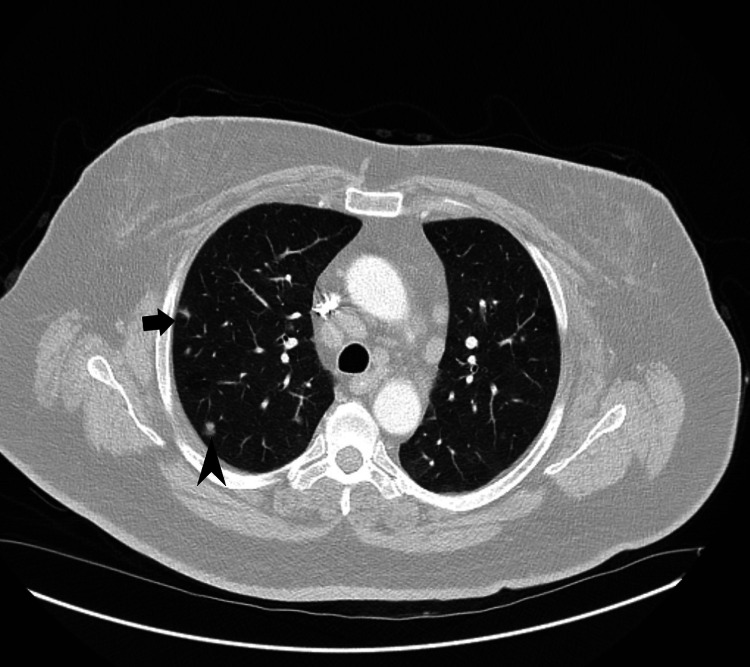
Transverse section of CT scan of the chest demonstrating multiple lung nodules in bilateral upper lobe distribution, including both cystic (block arrow) and solid (black arrowhead) lesions.

**Figure 3:**
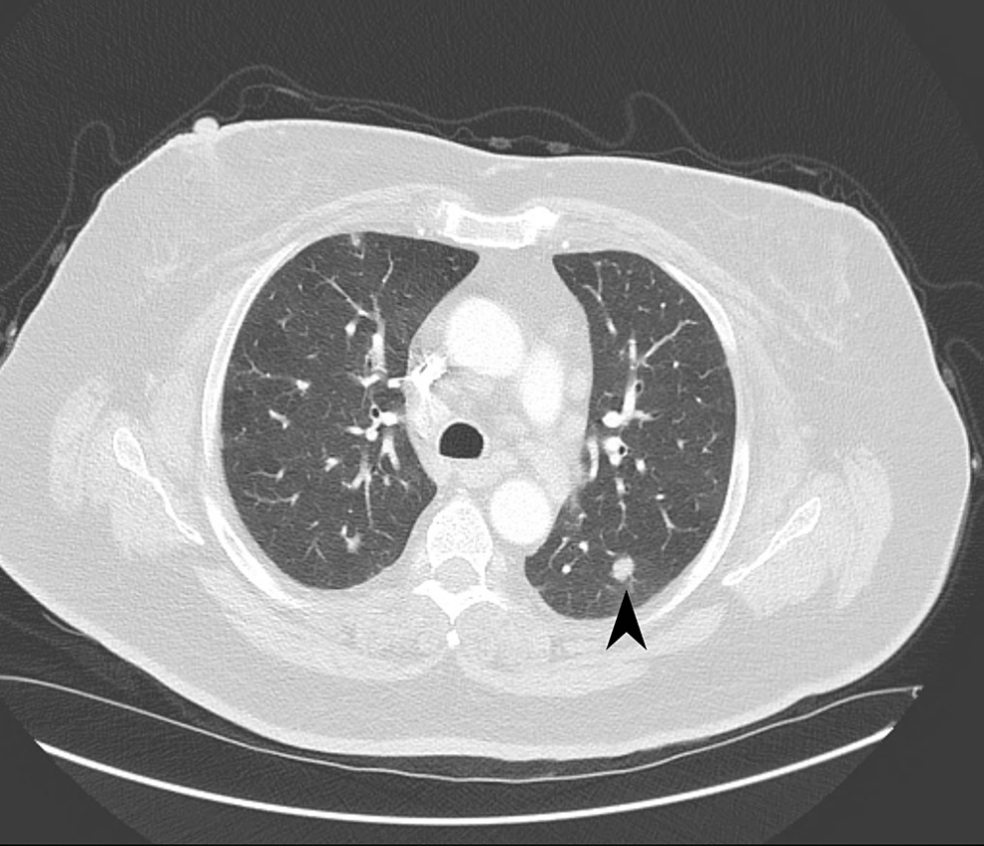
Transverse section of CT scan of the chest demonstrating large pulmonary nodule in the left posterior, upper lung field that was targeted for biopsy (black arrowhead).

**Figure 4:**
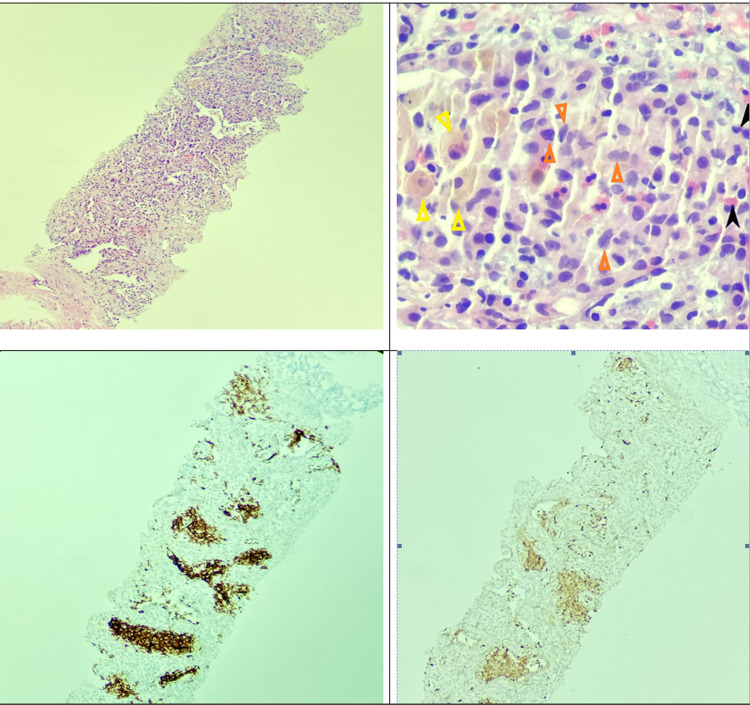
Upper left: low power view (x4) shows avolated lung tissue infiltrated by mixed chronic inflammatory infiltrate on lung nodule biopsy. Upper right: high-power view (x40) demonstrating pigmented macrophages suggestive of smoking (yellow triangles), histiocytes with cleaved nuclei (Langerhans cells) (orange triangles), eosinophils (black arrowheads), and plasma cells (red arrowheads) on lung nodule biopsy. Lower left: CD1a staining of lung nodule biopsy sample. Brown areas represent CD1a presence, highlighting aggregates of Langerhans cells and supporting the diagnosis. Lower right: S100 staining of lung nodule biopsy, highlighting aggregates of Langerhans cells and supporting the diagnosis.

The patient was sent for a lung cancer screening CT scan in January of 2021, during which multiple new pulmonary nodules were identified (Figures 1, 2). After extensive discussion with the patient, a decision was made to perform a biopsy of the largest lesion located in the left lung (Figure 3). Histopathological studies were consistent with PLCH (Figure 4). Additionally, a positron emission tomography (PET) scan was performed, which was found to be negative. At present, the disease is limited to strictly pulmonary involvement. Smoking cessation was emphasized, and follow-up imaging studies have been scheduled later in 2021 to follow progression or remission of her nodules.

## Discussion

Smoking cigarettes is a known risk factor for multiple pulmonary conditions, including COPD, emphysema, bronchitis, interstitial fibrosing lung disease, bronchiolitis, acute eosinophilic pneumonia, and Langerhans cell histiocytosis [2]. Initially, our patient had presented with mild dyspnea for a routine lung cancer screening CT scan, was found to have new lung nodules on chest imaging, and underwent further work-up further due to concerns for malignancy in view of her smoking history. She was ultimately diagnosed with biopsy-proven PLCH.

PLCH is a rare neoplastic condition that occurs almost exclusively in smokers, is commonest in a younger demographic (20-40 years of age), has no gender preference, and presents with multiple solid and/or cystic nodules in a primarily upper lobe distribution on chest imaging (Figures 1, 2) [2,3]. Definitive diagnosis requires biopsy and histopathological identification of eosinophilic granulomas with Langerhans cells present (CD1a, CD207, or S-100 positive), as with our patient (Figure 4) [2-5].

PLCH occurs as an isolated manifestation (i.e., without additional systems involvement) in 50%-70% of cases. Other sites commonly involved include bones, skin, and bone marrow. While less common, involvement may occur in the lymphopoietic systems, gastrointestinal tract, and central nervous system [3-5]. Interestingly, pulmonary involvement may be representative of one of the following three potential scenarios for the evolution of this condition: (1) occurrence as an isolated lesion, (2) pulmonary involvement after systemic disease, and (3) as an initial presentation of early disease that has the potential to progress. Symptoms can be vague and non-specific, including dyspnea, non-productive cough, hemoptysis, weight loss, chest pain, reduced exertional tolerance, and fever [3-5]. As many as 25% of patients can also present with incidental findings on chest imaging performed for either lung cancer screening or an alternate reason. Pneumothorax is an additional common entity that occurs in up to 45% of PLCH patients throughout the course of their disease and may be a presenting feature in 15%-20% of patients [2,3].

Our patient was dyspneic but did not have impaired pulmonary function testing and denied any constitutional symptoms (weight loss, appetite changes, night sweats, fever). She is currently still being monitored with serial imaging and follow-up for progression or resolution of incidental PLCH findings on imaging and subsequent biopsy. The literature demonstrates that as many as 30%-50% of patients will have spontaneous remission with cessation of tobacco exposure alone [3-5]. Additional treatment for persistent disease can include corticosteroids, chemotherapeutic agents (vinblastine, methotrexate, cyclophosphamide, cladribine, and etoposide), and, in extreme cases, lung transplantation [2-4]. A recently published review article also reviewed the use of novel agents such as mitogen-activated kinase inhibitors (trametinib, cobimetinib), BRAF kinase inhibitors (vemurafenib, dabrafenib), and tyrosine kinase inhibitors (imatinib) [3]. Despite the promise such agents hold, the data are still insufficient to make concrete recommendations, and further studies are necessary to assess the safety, efficacy, and morbidity associated with each agent.

## Conclusions

PLCH is a rare neoplastic syndrome of young smokers that may present with isolated pulmonary involvement or multi-system involvement. Patients can be asymptomatic or present with vague symptomatology. We presented the case of a 65-year-old female smoker who was found to have new lung nodules on lung cancer screening, underwent biopsy, and was diagnosed with PLCH thereafter. This particular patient is unique in that she falls well beyond the typical age demographic of 20-40 years, where this condition is most often expected. At present, there is no gold standard therapy, and further studies are needed to establish the safety, efficacy, and side effect profiles of novel agents being tested in patients refractory to smoking cessation and steroids.
